# Comparison of *Mycobacterium Tuberculosis* Genomes Reveals Frequent Deletions in a 20 kb Variable Region in Clinical Isolates

**DOI:** 10.1002/1097-0061(200012)17:4<272::AID-YEA48>3.0.CO;2-2

**Published:** 2000

**Authors:** Timothy B. L. Ho, Brian D. Robertson, G. Michael Taylor, Rory J. Shaw, Douglas B. Young

**Affiliations:** 1 Department of Infectious Diseases and Microbiology Imperial College School of Medicine Norfolk Place London W2 1PG UK; 2 Department of Respiratory Medicine Imperial College School of Medicine Norfolk Place London W2 1PG UK

## Abstract

The *Mycobacterium tuberculosis* complex is associated with a remarkably low level of structural gene polymorphism. As part of a search for alternative forms of genetic variation that may act as a source of biological diversity in *M. tuberculosis*, we have identified a region of the genome that is highly variable amongst a panel of unrelated clinical isolates. Fifteen of 24 isolates examined contained one or more copies of the *M. tuberculosis*-specific IS*6110* insertion element within this 20 kb variable region. In nine of the isolates, including the laboratory-passaged strain H37Rv, genomic deletions were identified, resulting in loss of between two and 13 genes. In each case, deletions were associated with the presence of a copy of the IS*6110* element. Absence of flanking tri- or tetra-nucleotide repeats identified homologous recombination between adjacent IS*6110* elements as the most likely mechanism of the deletion events. IS*6110* insertion into hot-spots within the genome of *M. tuberculosis* provides a mechanism for generation of genetic diversity involving a high frequency of insertions and deletions.


**Yeast**
*Yeast* 2000; **17**: 272–282.

**Research Article**

### Comparison of *Mycobacterium tuberculosis* genomes reveals frequent deletions in a 20 kb variable region in clinical isolates

Timothy B. L. Ho[1,2]*, Brian D. Robertson[1], G. Michael Taylor[1], Rory J. Shaw[2] and Douglas B. Young[1]
[1] *Department of Infectious Diseases and Microbiology, Imperial College School of Medicine, Norfolk Place, London W2 1PG, UK*
[2] *Department of Respiratory Medicine, Imperial College School of Medicine, Norfolk Place, London W2 1PG, UK*

**Correspondence to: T. B. L. Ho, Department of Infectious Diseases and Microbiology, Imperial College School of Medicine, Norfolk Place, London W2 1PG, UK. E-mail: t.ho@ic.ac.uk*

Received: 28 September 2000
Accepted: 28 September 2000

### Abstract

**The *Mycobacterium tuberculosis* complex is associated with a remarkably low level of structural gene polymorphism. As part of a search for alternative forms of genetic variation that may act as a source of biological diversity in *M. tuberculosis*, we have identified a region of the genome that is highly variable amongst a panel of unrelated clinical isolates. Fifteen of 24 isolates examined contained one or more copies of the *M. tuberculosis*-specific IS*6110* insertion element within this 20 kb variable region. In nine of the isolates, including the laboratory-passaged strain H37Rv, genomic deletions were identified, resulting in loss of between two and 13 genes. In each case, deletions were associated with the presence of a copy of the IS*6110* element. Absence of flanking tri- or tetra-nucleotide repeats identified homologous recombination between adjacent IS*6110* elements as the most likely mechanism of the deletion events. IS*6110* insertion into hot-spots within the genome of *M. tuberculosis* provides a mechanism for generation of genetic diversity involving a high frequency of insertions and deletions. Copyright © 2000 John Wiley & Sons, Ltd.**

**Keywords:** **gene deletions; insertion sequences; mycobacteria; strain variation; tuberculosis**

### Introduction

*Mycobacterium tuberculosis* is a highly successful human pathogen, infecting up to one-third of the global population and causing around two million deaths annually (Dye *et al.*, 1999). Successful pathogenesis is dependent on a combination of attributes, including the ability to resist killing by phagocytes, to establish a prolonged latent infection in healthy individuals and to exploit opportunities for active growth and aerosol transmission. Definition of the cellular and molecular mechanisms underlying these different stages of infection presents an important challenge in tuberculosis research. It has long been recognized that isolates of *M. tuberculosis* cultured from individual patients vary in their ability to survive under different conditions in the laboratory and to cause progressive infection in animals (Mitchison *et al.*, 1963; Ordway *et al.*, 1995). Similarly, epidemiological studies demonstrate heterogeneity in transmissibility and virulence amongst different isolates, as judged by rates of skin test conversion and onset of clinical disease amongst exposed contacts (Valway *et al.*, 1998; Rhee *et al.*, 1999). Investigation of genetic variation amongst *M. tuberculosis* isolates may provide insights into mycobacterial pathogenesis. By matching genetic differences between isolates with particular clinical or epidemiological patterns, it may be possible to identify genes that influence the biology of tuberculosis infection, and to understand the forces of microbial evolution that contribute to the success of this pathogen in a wide range of historical and geographical settings.

Comparative analysis of nucleotide sequences of a panel of structural genes has been used as an approach to investigate the extent of strain diversity

Copyright © 2000 John Wiley & Sons, Ltd.

amongst members of the *M. tuberculosis* complex (which includes the predominant veterinary pathogens *M. bovis* and *M. microti* as well as the major human pathogens *M. tuberculosis* and *M. africanum*) (Sreevatsan *et al.*, 1997). This approach uncovered a striking absence of allelic variation, generating findings consistent with a model in which *M. tuberculosis* evolved to its present form as a result of an evolutionary 'bottleneck' around 15 000–20 000 years ago. In contrast, a more extensive diversity has been revealed by whole genome comparisons within the *M. tuberculosis* complex (Behr *et al.*, 1999; Gordon *et al.*, 1999). A series of chromosomal deletions were identified, with around 100 of the 4000 genes present in *M. tuberculosis* being absent from the genome of *M. bovis* isolates. A further 30 genes were found to have been selectively lost during the years of *in vitro* culture of *M. bovis* that led to generation of the current set of attenuated isolates used for preparation of the BCG (bacille Calmette Guérin) vaccine. These genetic differences may underlie variations in host specificity of the pathogenic isolates, and are most probably responsible for attenuation of the vaccine strains (Behr *et al.*, 1999).

Amongst isolates of *M. tuberculosis* itself, genome variation is evident from patterns revealed by pulsed-field gel electrophoresis (Zhang *et al.*, 1992). Factors that contribute to this genetic heterogeneity are known to include differences in the copy number and location of an *M. tuberculosis*-specific insertion sequence (IS*6110*), variations amongst a set a polymorphic GC-rich sequences (PGRS) present in genes encoding a family of proteins with unknown function, and short variable sequences interspersed within an apparently non-coding region of the genome characterized by the presence of a series of direct repeat elements (the 'DR region') (van Embden *et al.*, 1993; Chaves *et al.*, 1996; Kamerbeek *et al.*, 1997). These markers have been extensively employed for strain-typing – proving particularly useful in identification of clusters associated with transmission chains (Small *et al.*, 1994) – but their potential contribution to variation in phenotypic properties of the different isolates has received less attention.

The present study was initiated with the aim of identifying sites of genetic variation amongst *M. tuberculosis* isolates that may contribute to differences in the biological properties of the organisms. To identify candidate loci, the genome of *M. tuberculosis* H37Rv – a well-characterized isolate for which the complete sequence has been determined (Cole *et al.*, 1998) – was analysed for evidence of IS*6110*-mediated gene disruption. There are 16 copies of the IS*6110* insertion element in the H37Rv; five of these are inserted within predicted open reading frames (ORFs) (Sampson *et al.*, 1999). One of the IS*6110* insertions, encoded by Rv1756c and Rv1757c, is associated with truncation of the N-terminal region of two ORFs arranged in opposite orientations and predicted to encode phospholipase C (*plcD*) and cutinase enzymes (Rv1755c and Rv1758, respectively). The N-terminal portions that are missing from the two H37Rv ORFs are present in the genome of a second well-characterized clinical isolate, CDC1551 (Valway *et al.*, 1998), together with three additional intervening ORFs which are not found in H37Rv (Figure 1). The missing genes are also present in the laboratory-attenuated strain H37Ra (Brosch *et al.*, 1999) and correspond to the RvD2 deletion initially described as being present in *M. bovis* but absent from H37Rv (Gordon *et al.*, 1999). In the present study, we have analysed this region in a panel of clinical isolates of *M. tuberculosis*, and have identified it as an area of extensive genetic diversity resulting from frequent insertion and deletion events.

### Materials and methods

### Sequence comparison

The nucleotide sequence of *M. tuberculosis* H37Rv from the Sanger Centre (**http://www.sanger.ac.uk/Projects/M_tuberculosis/**) was compared to the sequence of cosmid Y28 (Accession No. Z95890) and the incomplete and unannotated nucleotide sequence of *M. tuberculosis* CDC1551 (The Institute for Genome Research, **http://www.tigr.org**, October 1998 version) using the BLAST 2.0 programme at the National Center for Biotechnology Information (NCBI, **http://www4.ncbi.nlm.nih.gov/BLAST**). The Genemark programme (European Bioinformatics Institute, **http://www2.ebi.ac.uk/genemark**) was then used to predict open reading frames (ORFs) of sequences present in the CDC1551 strain. These ORFs were compared against the EMBL databases, using BLAST to look for similar sequences. Subsequently, partial annotations for ORFs of the CDC1551 strain became available (**http://www.**

Copyright © 2000 John Wiley & Sons, Ltd.

Table 1. PCR primers and conditions

| Target region | Primer name | Primer sequence (5′ to 3′) | Annealing temp. (°C) | Extension time | Predicted product size (bp) |
|---|---|---|---|---|---|
| Standard amplifications (*Taq* polymerase) | | | | | |
| Probe for IS*6110* | INS1 | CGT GAG GGC ATC GAG GTG GC | 66 | 40 s | 226 |
| | INS2 | GCG TAG GCG TCG GTG ACA AA | | | |
| Rv1754c fragment | PR1 | TTC ATA CCG TTG GTG TAG AGC | 59 | 15 s | 224 |
| | PR2 | GGA CTC ATG GTT CCA ATA GG | | | |
| *plcD* fragment | P1 | CAG CGA AGT TGA ACG TTG AC | 65 | 2 min 10 s | 592 |
| | P2 | CTT ACT TAC GGC TCG CTT GT | | | |
| Cutinase fragment | C1 | ACC ACG GAT TTC CCG ACA GC | 67 | 30 s | 411 |
| | C2 | AAA CAC TGC GGC CTG CTC G | | | |
| *plcD* to cutinase | P2 | CTT ACT TAC GGC TCG CTT GT | 57 | 2 min | 1556 |
| | C3 | GAT GGC CGG TAT TTA CGA C | | | |
| Rv1766-1767 fragment | R1 | GTT GAA GGA ATG CGT GTC C | 59 | 16 s | 237 |
| | R2 | GGG AAT CTG GTG ACG TAG A | | | |
| MT1801 fragment | M3 | AGA ATT ACT TTC AGG CTC TGG A | 58 | 15 s | 178 |
| | M4 | CCA TCC CAT AGC CAC GAA T | | | |
| Rv1760 fragment | V1 | ATG GAG CGA CTA AGC GGA CT | 64 | 15 s | 186 |
| | V2 | GCG AGC TTC ATC CGA AAT T | | | |
| Extended amplifications (eLONGase mix) | | | | | |
| *plcD* to *mmpl4* | NP2 | ACA AGC GAG CCG TAA GTA AG | 60 | 6 min | 5690 |
| | M2 | TTT GGT GAG ACA AAA TAG TCC A | | | |
| *mmpl4* to Rv1758 (cutinase) | M1 | GAC TAT TTA CGC GAA CTT GCC | 64 | 3 min | 2797 |
| | C3 | GAT GGC CGG TAT TTA CGA C | | | |
| Rv1754c to Rv1758 | PR3 | AAT GCG GAT ATC AGT GGA C | 64 | 4 min 30 s[1] | 2961 |
| | C3 | GAT GGC CGG TAT TTA CGA C | | | |
| Rv1754c to Rv1767 | PR3 | AAT GCG GAT ATC AGT GGA C | 59 | 6 min 40 s[2] | 13939 |
| | R2 | GGG AAT CTG GTG ACG TAG A | | | |
| Rv3324c to Rv3328c | MolyF | TTC ATC AAG GTG GGT AAG C | 58 | 6 min 11 s | 3464 (Rv) |
| | MolyR | AGG TGG TCC GGT TCA TAC T | | | 6170 (CDC) |

[1]7 μl eLONGase buffer A, 3 μl eLONGase buffer B, 2.5 μl dimethyl sulphoxide.

[2]2.5 μl Dimethyl sulphoxide.

**tigr.org/tdb/CMR/gmt/htmls/SplashPage.html**, July 1999 version). These are prefixed in the text with 'MT' followed by the ORF number. In contrast, ORFs referring to the H37Rv strain are prefixed with 'Rv'.

### Clinical isolates and DNA extraction

Clinical isolates of *M. tuberculosis* were taken from cultures derived from clinical specimens isolated in the Mycobacteria Laboratory at St Mary's Hospital, Paddington, London, UK. All of the patients were infected with drug-sensitive strains, and responded to conventional therapy. A culture of the clinical isolate CDC1551 was kindly provided by Dr Jack Crawford at the Centers for Disease Control and Prevention, Atlanta, GA, USA. Cultures were grown in Middlebrook 7H9 medium, supplemented with albumin, dextrose and catalase (Difco). Genomic DNA was extracted from cultures in the exponential phase of growth by standard phenol–chloroform extraction, as described previously (Goyal *et al*., 1997), and made up in HPLC grade water at a concentration of approximately 50 μg/ml.

### Amplification by polymerase chain reaction (PCR)

All reactions were carried out using a 48-well Hybaid Touchdown PCR thermocycler (Hybaid). A standard 25 μl reaction mixture was used containing 25 pmol of each primer in 1 μl, 2.5 μl of a 2 mM deoxyribonucleotide mix, 2.5 μl 10 × reaction buffer (Promega), 2 μl 5 mM $MgCl_2$ and 1.5 units *Taq* polymerase (Promega). To this was added 3 μl

Copyright © 2000 John Wiley & Sons, Ltd.

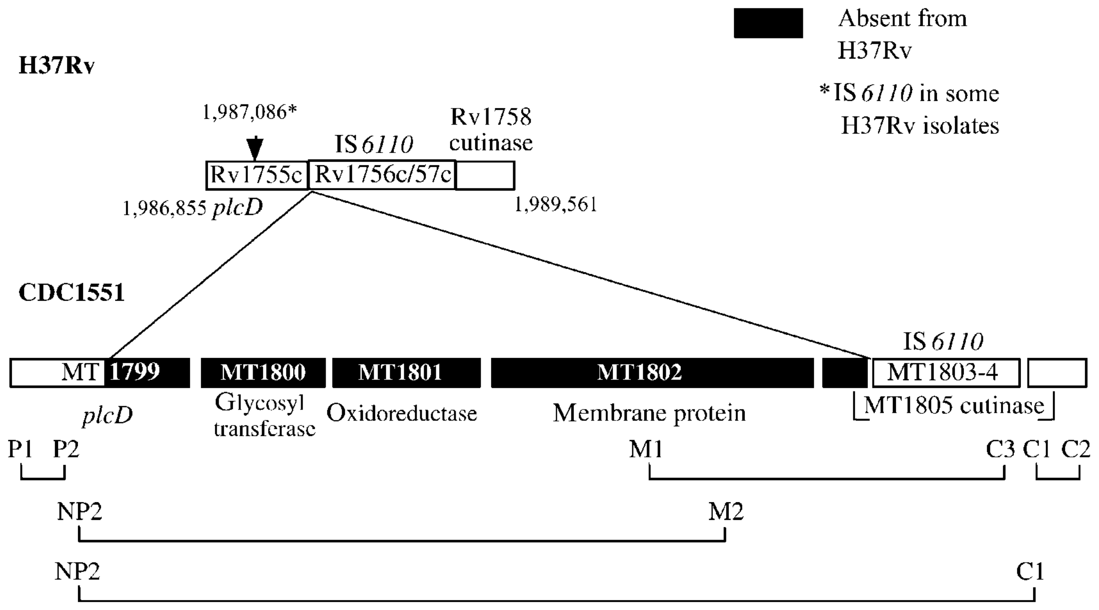

**Figure 1.** Organization of the *plcD*-cutinase region in *M. tuberculosis* isolates H37Rv and CDC1551

DNA solution and the volume made up to 25 µl with HPLC grade water (Sigma-Aldrich). Thermocycling parameters were as follows: 1 cycle at 94°C for 30 s, held at 85°C while the *Taq* enzyme was added, followed by 35 cycles of 94°C for 30 s, 30 s at the specified annealing temperature, 72°C for the specific extension time required, followed by a final cycle at 72°C for 3 min.

For predicted products greater than 2 kb, PCR was performed using the eLONGase enzyme mix (Gibco/BRL). Each 50 µl PCR reaction mixture contained 25 pmol of each primer, 5 µl 2 mM deoxyribonucleotide mix, 5 µl 5 × eLONGase Buffer A, 5 µl 5 × eLONGase Buffer B (unless otherwise stated) and 2 µl eLONGase enzyme mix. To this was added 3 µl DNA solution and the volume made up to 50 µl with HPLC grade water (Sigma-Aldrich). Thermocycling parameters were as follows: 1 cycle at 94°C for 30 s, held at 85°C while the enzyme mix was added, followed by 35 cycles of 94°C for 30 s, 30 s at the specified annealing temperature, 68°C for the specific extension time required, followed by a final cycle at 68°C for 10 min. Details of primer pairs, specific annealing temperatures and extension times are listed in Table 1.

PCR products were examined by routine gel electrophoresis performed on 1% (w/v) agarose gels in TAE buffer. Products for sequencing were excised using a sterile scalpel blade and purified using the GENECLEAN II kit (Bio 101), according to the manufacturer's instructions.

### Dot-blot hybridization

A 2 µl sample of PCR product amplified using the M1/C3 primer pair was denatured by heating at 94°C for 2 min, snap-cooled on ice, and applied to a Hybond $N^+$ membrane (Amersham) and air-dried. The DNA was cross-linked to the membrane using a UV cross-linker (Stratagene). As a probe, 1 µg INS1/INS2 amplicon from IS*6110* was labelled for 16 h using the DIG High Prime DNA labelling kit (Boehringer-Mannheim), in accordance with the manufacturer's instructions. The membrane was prehybridized using DIG Easy Hyb (Boehringer-Mannheim) for 1 h at 37°C. The probe was denatured by boiling for 5 min and snap-cooled in ice-water. The probe was then added to the hybridization bottle and incubated overnight at 37°C. Post-hybridization washes and chemiluminescence detection steps were carried out according to the manufacturer's protocol, using the Boehringer-Mannheim Detection kit.

Copyright © 2000 John Wiley & Sons, Ltd.

Table 2. Clinical isolates of *Mycobacterium tuberculosis*

| Patient | Disease site | Sex | Smear status | Racial origin | Genotype* |
|---|---|---|---|---|---|
| TH1 | Lymph node | Male | Negative | Sudanese | D |
| TH2 | Lymph node | Female | Negative | Philippino | U |
| TH3 | Lymph node | Male | Negative | Somalian | U |
| TH4 | Lymph node | Female | Negative | Somalian | D |
| TH5 | Lymph node | Male | Negative | Somalian | U |
| TH6 | Lymph node | Male | Negative | Chinese | U |
| TH7 | Lymph node | Female | Negative | Saudi Arabian | D |
| TH8 | Chest wall | Male | Negative | Somalian | I |
| TH9 | Spine | Female | Positive | Caucasian (Albanian) | D |
| TH10 | Sputum | Male | Positive | Moroccan | I |
| TH11 | Sputum | Male | Positive | Afro-Caribbean | I |
| TH12 | Sputum | Male | Negative | Somalian | D |
| TH13 | Sputum | Female | Negative | Indian | D |
| TH14 | Sputum | Male | Negative | Indian | U |
| TH15 | Sputum | Male | Positive | Algerian | D |
| TH16 | Sputum | Female | Positive | Eritrean | U |
| TH17 | Sputum | Female | Positive | Somalian | U |
| TH18 | Sputum | Female | Positive | Indian | I |
| TH19 | Sputum | Male | Negative | Indian | U |
| TH20 | Sputum | Male | Positive | Bangladeshi | U |
| TH21 | Sputum | Male | Positive | Caucasian (Ukrainian) | D |
| TH22 | Sputum | Male | Negative | Caucasian (British) | I |

*Genotype within 20 kb hypervariable island. U = unmodified; I = insertional inactivation; D = gene deletion.

### Automated DNA sequencing

Cycle sequencing of PCR products was performed using a Hybaid Touchdown PCR machine and dichlororhodamine dye terminator ready reaction mixture (PE Biosystems) in accordance with the manufacturer's protocol. Subsequent analysis was performed on an ABI 310 Genetic analyser (PE Biosystems). The primers used for sequencing were: PLC2, 5′ CAC TAG CCG AGA CGA TCA AC 3′; and PLC3, 5′ CGC CTG GCG CAC CCA CTT AC 3′.

### Results

### Analysis of *plcD*-cutinase region

To assess the frequency of diversity in the *plcD*-cutinase region (Figure 1), a panel of 22 unrelated clinical isolates of *M. tuberculosis* was collected from patients attending the tuberculosis clinic at St. Mary's Hospital. The isolates were selected to represent a range of disease presentations, including involvement of pulmonary and extrapulmonary sites, and smear-positive and smear-negative specimens at the time of diagnosis (Table 2). Together with the well-characterized H37Rv and CDC1551 isolates, this panel was screened using a series of PCR assays to characterize the *plcD* gene, the cutinase gene and the intervening region.

### Phospholipase C

A PCR assay was set up using primers P1 and P2 to amplify a 0.6 kb fragment from the *plcD* gene described in the *M. tuberculosis* H37Rv genome sequence (Cole *et al*., 1998). Initial standardization of this assay, using H37Rv cultures from our laboratory stock, generated a larger than expected product of approximately 2 kb. Analysis of this fragment demonstrated the presence of an additional copy of the IS*6110* insertion element interrupting the C-terminal portion of the protein. A similar insertion was previously reported during analysis of cosmid clone Y28 at an intermediate stage of the H37Rv sequencing project (Z95890, EMBL release 52, May 1997), but was not found in the BAC library used to complete the definitive H37Rv genome sequence (Cole *et al*., 1998). Analysis of the nucleotide sequence in our local H37Rv isolate identified the point of insertion at

Copyright © 2000 John Wiley & Sons, Ltd. 

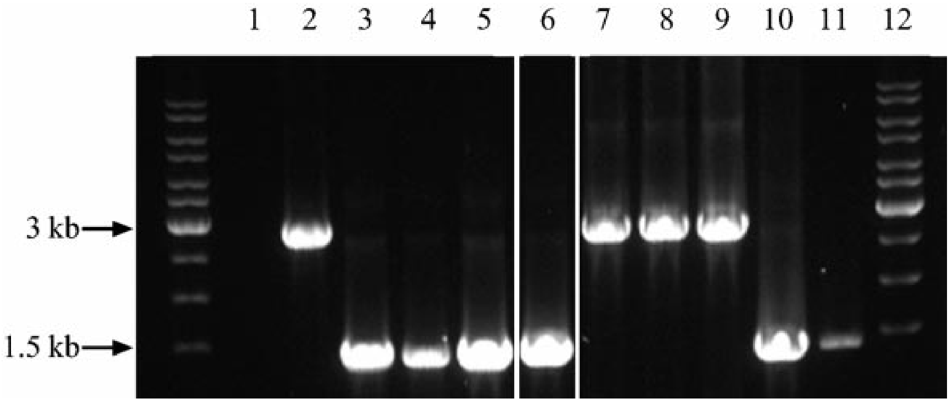

**Figure 2.** Gel electrophoresis of PCR products generated with M1/C3 primers.Three different patterns were found following PCR amplification of a region spanning genes encoding membrane protein MT1801 and cutinase, characterized by the presence of a 3.0 kb fragment, a 1.5 kb fragment, or the absence of any detectable product. Lane 1, negative control; 2, CDC1551; 3, TH6; 4, TH16; 5, TH20; 6, TH14; 7, TH22; 8, TH8; 9, TH10; 10, TH19; 11, TH3; 12, 1 kb ladder position 1 987 086 – a location identical to that described in the Y28 sequence. This finding demonstrates a degree of heterogeneity in the pattern of IS*6110* insertions in the *plcD*-cutinase region amongst H37Rv cultures derived from a single initial source. Application of the PCR assay to the panel of clinical isolates identified one further sample (TH11) with an IS*6110* insertion affecting the C-terminal region of phospholipase C. The point of insertion in this case was at a position 371 bp downstream from the H37Rv insertion. Amongst the other isolates, 15 generated the expected 0.6 kb fragment (TH2, 3, 5, 6, 8, 9, 10, 14, 16, 17, 18, 19, 20, 22 and CDC1551), while the remaining seven isolates produced no detectable PCR product (TH1, 4, 7, 12, 13, 15, 21) (Table 3).

### Cutinase

A second PCR assay was established using primers C1 and C2 to amplify a 0.4 kb fragment identified in the H37Rv cutinase sequence. In this case, the expected product was obtained from 16 of the isolates. Of the remaining six isolates that did not amplify (TH1, 7, 9, 12, 13, 21), all but one (TH9) had also failed to produce a result with the *plcD* assay.

Three PCR assays were then used to characterize the regions encoding the N-terminal portions of the phospholipase and cutinase enzymes, together with the region linking the two genes. Primers spanning from the *plcD* to the cutinase genes (NP2/C3) amplified a 1.5 kb fragment from H37Rv (corresponding to the IS*6110* insertion element recorded in the genome sequence), but failed to generate a product with CDC1551 or with any of the clinical isolates. Products were obtained, however, when the reaction was redesigned to amplify two shorter products, using primers based on the sequence of MT1802, an ORF absent from H37Rv but lying between *plcD* and the cutinase gene in CDC1551 (Figure 1). The isolates could be divided into three groups on the basis of results with primers M1 and C3, amplifying the region from MT1802 to the cutinase (Figure 2). Four isolates, TH8, 10, 18 and 22, generated a 3 kb product (identical to that predicted from the CDC1551 sequence). A 1.5 kb product was obtained from a further 10 isolates (TH2, 3, 5, 6, 11, 14, 16, 17, 19, 20), while the remaining eight isolates (TH1, 4, 7, 9, 12, 13, 15, 21) resembled H37Rv in generating no product. Hybridization experiments identified the presence of the IS*6110* insertion element in each of the 3 kb products. The point of insertion was determined by sequencing the flanking regions at both ends of the IS*6110* element. In two of the isolates, TH8 and TH22, the insertion was located within the cutinase gene at a location identical to that observed in the H37Rv and CDC1551 sequences (position 1 989 056), although in TH22 the orientation of IS*6110* was opposite to that seen in the characterized strains. In the two other isolates (TH10 and 18), the insertion occurred at a point 22 bp further downstream. Primers NP2 and M2 – designed to amplify the region from *plcD* to MT1802 –

Copyright © 2000 John Wiley & Sons, Ltd. 

Table 3. Genetic diversity within the variable island

| Isolate number | Size of PCR product (kb) *plcD* (P1–P2) | Cutinase (C1–C2) | Cut/MT1802 (M1–C3) | IS*6110* insertion | Position | Deletion size (kb) |
|---|---|---|---|---|---|---|
| Unmodified isolates | | | | | | |
| TH2 | 0.6 | 0.4 | 1.5 | None | | |
| TH3 | 0.6 | 0.4 | 1.5 | None | | |
| TH5 | 0.6 | 0.4 | 1.5 | None | | |
| TH6 | 0.6 | 0.4 | 1.5 | None | | |
| TH14 | 0.6 | 0.4 | 1.5 | None | | |
| TH16 | 0.6 | 0.4 | 1.5 | None | | |
| TH17 | 0.6 | 0.4 | 1.5 | None | | |
| TH19 | 0.6 | 0.4 | 1.5 | None | | |
| TH20 | 0.6 | 0.4 | 1.5 | None | | |
| Insertion isolates | | | | | | |
| TH8 | 0.6 | 0.4 | 3.0 | Cutinase | 1 989 056 | |
| TH10 | 0.6 | 0.4 | 3.0 | Cutinase | 1 989 078 | |
| TH11 | 2.0 | 0.4 | 1.5 | *plcD* | 1 987 457 | |
| TH18 | 0.6 | 0.4 | 3.0 | Cutinase | 1 989 078 | |
| TH22 | 0.6 | 0.4 | 3.0 | Cutinase | 1 989 056 | |
| CDC1551 | 0.6 | 0.4 | 3.0 | Cutinase | 1 989 056 | |
| Deletion isolates | | | | | | |
| TH1 | ND | ND | ND | *plcD* | 1 987 086 | 19.2 |
| | | | | ISB9 | 1 998 791 | |
| TH4 | ND | 0.4 | ND | Rv1754c | 1 986 620 | 9.3 |
| | | | | Cutinase | 1 989 079 | |
| TH7 | ND | ND | ND | *plcD* | 1 987 086 | 19.2 |
| | | | | ISB9 | 1 998 791 | |
| TH9 | 0.6 | ND | ND | Cutinase* | 1 975 232 | 8.6 |
| | | | | MT1812* | 1 983 811 | |
| TH12 | ND | ND | ND | *plcD* | 1 987 455 | 18.9 |
| | | | | ISB9 | 1 998 847 | |
| TH13 | ND | ND | ND | *plcD* | 1 987 455 | 18.8 |
| | | | | ISB9 | 1 998 748 | |
| TH15 | ND | 0.4 | ND | Rv1754c | 1 986 616 | 9.3 |
| | | | | Cutinase | 1 989 081 | |
| TH21 | ND | ND | ND | Rv1754c | 1 986 639 | 19.5 |
| | | | | ISB9 | 1 998 621 | |
| H37Rv | 0.6 | 0.4 | ND | *plcD* | 1 987 746 | 6.8 |
| | | | | Cutinase | 1 989 056 | |
| | | | | Rv1763 | 1 996 150 | 0.7 |
| | | | | Rv1764 | 1 997 411 | |
| H37Rv (St Mary's) | 2.0 | 0.4 | ND | *plcD* insertion | 1 987 086 | |

*Refers to CDC1551 gene positions. All other positions are relative to H37Rv. ND: No detectable product.

generated a 5.7 kb fragment from CDC1551 and from the 14 clinical isolates positive in the *plcD* PCR assay.

The results of the above analyses (summarized in Table 3) demonstrated that in nine of the isolates (TH2, 3, 5, 6, 14, 16, 17, 19, 20) the intact *plcD* and cutinase genes were present together with the three intervening genes in the absence of any IS*6110* insertion. In a further four isolates (TH8, 10, 18, 22) and CDC1551, all of the genes were present, but in each case the cutinase gene was interrupted by IS*6110* inserted in either orientation at one of two sites. In one isolate (TH11), the genes were intact with the exception of an insertion in *plcD*. Characterization of the *plcD*-cutinase region in the remaining eight isolates (TH1, 4, 7, 9, 12, 13, 15, 21) was frustrated by the absence of either or both of the *plcD* and cutinase genes.

Copyright © 2000 John Wiley & Sons, Ltd. 

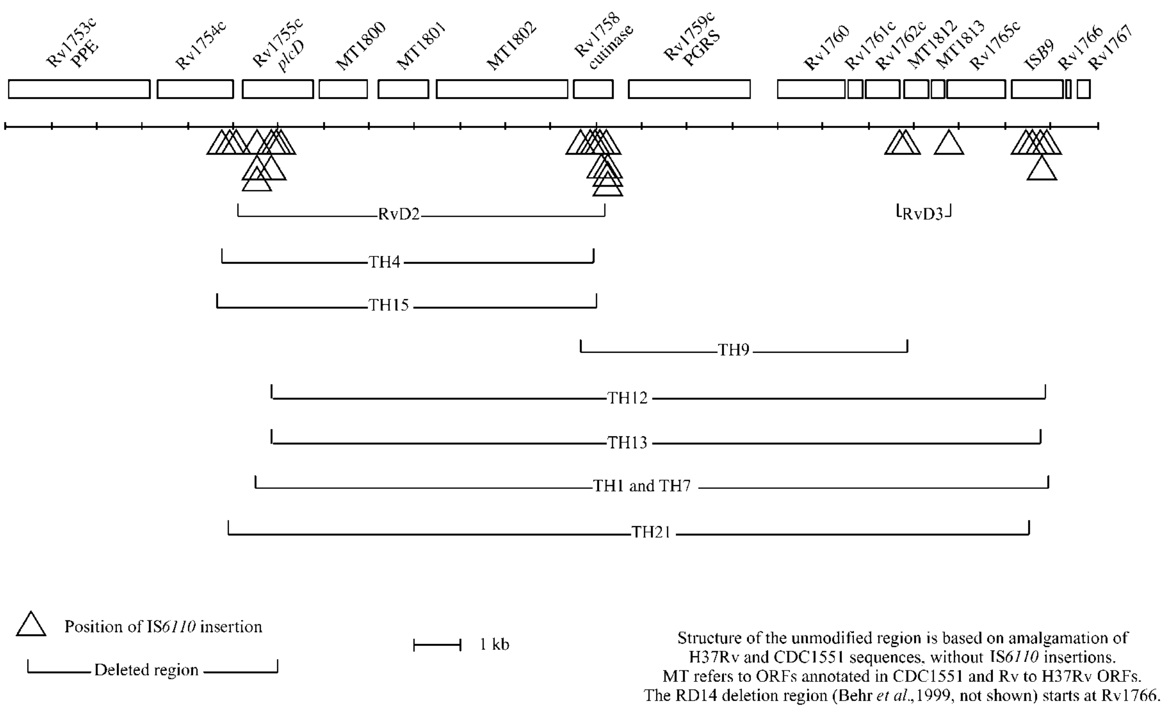

**Figure 3.** Mapping of insertions and deletions within the 20 kb variable island

### Mapping of novel deletion events

### Upstream genes

To characterize the remaining eight clinical isolates, a series of PCR primers were designed to test for the presence of genes lying outside the *plcD*-cutinase region (Figure 3). All eight isolates generated positive results using a PCR assay (primers PR1/PR2) designed to amplify a 0.2 kb fragment from the ORF directly upstream of *plcD* (Rv1754c, encoding a proline-rich protein). In the case of the two isolates which were positive for the cutinase gene (TH4, 15), primers based on cutinase and Rv1754c amplified 1.8 kb fragments, each containing a copy of the IS*6110* insertion element. Sequence analysis identified adjacent, but distinct, insertion points in the two isolates (Table 3). In each case the IS*6110* element joined Rv1754c directly to the cutinase, and was associated with loss of the intervening genes.

### Downstream genes

Positive results were generated from the remaining six cutinase-negative isolates (TH1, 7, 9, 12, 13, 21) using PCR primers R1 and R2 directed towards a 0.2 kb fragment overlapping downstream ORFs Rv1766 and Rv1767 (Figure 3). Further amplification reactions using R2 in combination with primers from Rv1754c (PR3) and MT1802 (M1) generated products from each of the isolates. Each of the products contained a copy of the IS*6110* insertion sequence, and in each case the insertion was associated with loss of intervening genes. In four of the isolates (TH1, 7, 12, 13), IS*6110* linked a portion of the *plcD* gene to an intergenic region annotated as having homology to a possible insertion element (IS*B9*). In two of these isolates (TH1, 7) the *plcD* insertion was at the same point as the additional C-terminal insertion identified in cosmid Y28 and in our local H37Rv culture; in the other two isolates (TH12, 13) the insertion point was 369 bp closer to the 5′ end of the gene. In a fifth isolate, TH21, the IS*6110* insertion element linked Rv1754c directly to the IS*B9* region, with the loss of all of the intervening ORFs, while the final isolate (TH9) had an insertion joining the cutinase gene to MT1812, an ORF present in CDC1551 but absent from H37Rv (Figure 3).

Copyright © 2000 John Wiley & Sons, Ltd.

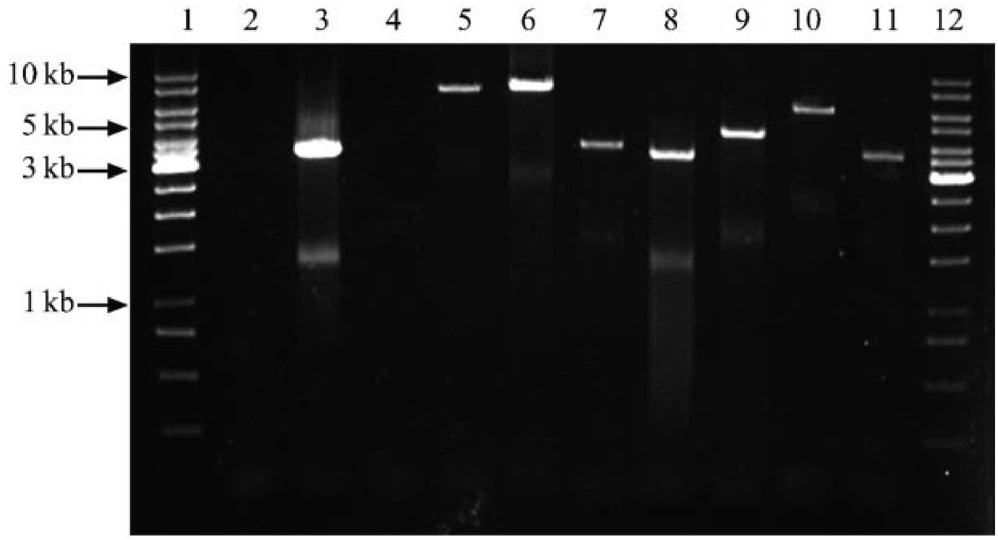

**Figure 4.** Genetic diversity in the IS*1547* region.PCR amplification using primers spanning Rv3324c to Rv3328c revealed extensive polymorphism amongst the panel of *M. tuberculosis* clinical isolates, with products ranging in size from 3 to 10 kb. Lane 1, 1 kb ladder; 2, negative control; 3, H37Rv; 4, negative control; 5, TH19; 6, TH15; 7, TH12; 8, TH8; 9, TH4; 10, TH2; 11, TH1; 12, 1 kb ladder

### IS*6110* and gene deletion

A potential mechanism to account for the presence of IS*6110* elements in association with gene deletion has been proposed by Fang *et al.* (1999). This involves a homologous recombination event between adjacent copies of IS*6110*, and is accompanied by loss of the characteristic three or four nucleotide direct repeat flanking IS*6110* elements involved in the normal insertion process. To evaluate the possible contribution of this mechanism to the results described above, sequences flanking each side of the IS*6110* insertions were determined. In each case, when IS*6110* was linked to the loss of genes, the flanking direct repeat was absent, consistent with the recombination-mediated deletion mechanism.

Screening of sequences flanking IS*6110* insertion elements in the H37Rv genome (Cole *et al.*, 1998) identifies four copies lacking the trinucleotide repeat. The unflanked copies have each been shown to be associated with deletions from H37Rv when compared to other *M. tuberculosis* and *M. bovis* isolates and have been annotated as RvD2 to RvD5, respectively (Brosch *et al.*, 2000). One is involved in the *plcD*-cutinase deletion (RvD2), and a second is associated with the deletion of MT1812 and MT1813 (RvD3), illustrated in Figure 3. A third unflanked copy occurs between two PPE genes (RvD4) and the final example, Rv3325-3326, corresponds to the IS*1547*-associated copy described by Fang *et al.* (1999) as characteristic of the variable *ipl* locus (RvD5). A PCR assay (primers MolyF and MolyR) designed to amplify this locus generated a range of products from the different isolates (Figure 4), demonstrating that this region is also variable amongst the panel of isolates included in the present study.

### Discussion

Analysis of a 20 kb region of the *M. tuberculosis* chromosome in a panel of 22 clinical isolates has revealed surprisingly extensive genetic diversity. Insertion and deletion events were identified in more than half of the isolates, with effects ranging from disruption of individual open reading frames to loss of as many as 13 genes. Variability within this region is associated with a high frequency of IS*6110* insertions. We have identified a total of 18 discrete insertion sites, clustered predominantly in three subregions comprising *plcD* and the adjacent Rv1754c, the cutinase gene, and an IS*B9*-like element. A similar high frequency of IS*6110* insertion events within this region was observed in a recent study of South African isolates (Sampson *et al.*, 1999). Where IS*6110* is associated with loss of

Copyright © 2000 John Wiley & Sons, Ltd. 

genes, the absence of flanking direct repeat elements strongly suggests that homologous recombination between adjacent copies of the insertion element has been the cause of the deletion event. No clinical isolate was found to have a double IS*6110* insertion in this region. The presence of two proximally related IS elements may lead to an unstable conformational structure of the genome, thus precipitating homologous recombination. The presence of other IS*6110*-associated deletion regions in *M. tuberculosis* H37Rv (RvD3–RvD5) (Brosch *et al.*, 2000) suggests that the 20 kb region is not unique and probably represents an example of a general phenomenon. These findings identify gene deletion as an important source of genetic diversity amongst clinical isolates of *M. tuberculosis*.

Two important questions arise from this study. Firstly, is the variable island unique, or is it representative of a general mechanism of genetic diversity in *M. tuberculosis*? Secondly, does the loss of genes – by insertion or by deletion – have a significant effect on the biological properties of the different isolates?

Diversity within the 20 kb region would appear to be the result of its status as a 'hot-spot' for IS*6110* insertion. Other IS*6110* hot-spots have been described (Fomukong *et al.*, 1997; Fang *et al.*, 1997; Kurepina *et al.*, 1998) and two mechanisms may account for their occurrence. First, insertion at a particular position may confer some selective advantage which is then preserved during subsequent strain diversification. Alternatively, local structural features may make certain regions of the genome particularly susceptible to insertion events. The frequent finding of clustered insertions, as well as insertions in similar locations but with different orientation, is indicative of the existence of regions prone to multiple insertion events as envisaged in the second mechanism. Similarly, variation in insertion patterns amongst contemporary H37Rv isolates, as reflected in the present study and in the comparison with H37Ra (Brosch *et al.*, 1999), is consistent with a high frequency of IS*6110* activity associated with the *plcD* gene, rather than immortalization of a single rare event. Interestingly, the 20 kb region overlaps with the RD14 BCG Pasteur deletion described by Behr *et al.* (1999). The absence of IS*6110* in this case suggests that this region may also be susceptible to alternative mechanisms of deletion.

Turning to the biological consequences of genotypic variation in the *plcD*-cutinase region; recovery of the deletion strains from patients with active tuberculosis demonstrates that they retain the ability to cause disease. An influence of the deletion genes on the course of infection is not excluded, however. Phospholipase C has been identified as a virulence factor for several bacterial pathogens (Titball, 1993) whilst cutinases, although principally associated with the ability of fungi to penetrate the cutin layers of plants (Schafer, 1993), may have been adapted for hydrolysis of some related polymer during mammalian infection. Both genes are found as part of multicopy families. Interestingly, three of the phospholipase C genes are deleted from *M. bovis*, leaving *plcD* as the sole source of enzyme activity in the case of bovine infection (Behr *et al.*, 1999; Gordon *et al.*, 1999) and some clinical isolates of *M. tuberculosis* demonstrate evidence of polymorphism in this region (Vera-Cabrera *et al.*, 1997). The different homologues could provide some level of diversity in terms of precise substrate specificity, or may simply contribute to an increase in the overall amount of enzyme that can be produced. The loss of one or more copies could therefore affect the balance of the host–pathogen interaction. Other ORFs identified in the variable region encode protein products sharing homology with glycosyl transferases (MT1800), oxidoreductases (MT1801) and enzymes involved in the synthesis of antibiotics (Rv1760).

The high prevalence of deletion events suggest that they may be subject to positive selection, either by some specific influence on the process of infection, as discussed above, or simply as a result of removal of genes no longer required by the bacteria. If this is the case, it would be anticipated that the deletion strains represent a later stage of *M. tuberculosis* evolution, as compared to those having an intact complement of genes within the variable region. Sreevatsan *et al.* (1997) have proposed an evolutionary lineage for *M. tuberculosis* isolates based on specific nucleotide substitutions in codons of the *katG* and *gyrA* genes. Examination of these sequences within the present panel failed to demonstrate any correlation. Deletion and non-deletion isolates were distributed amongst both early and late Sreevatsan groups (Sales MPU, Ho TBL, Taylor GM, unpublished observations). Thus, if deletion events do represent an evolutionary progression, it is not one that is coordinated with the sequence of *katG*/*gyrA* substitutions.

Copyright © 2000 John Wiley & Sons, Ltd. 

The very limited panel employed in the present study did not reveal any obvious association between genotype and clinical presentation or geographical origin. However, a more extensive analysis – such as that described recently by Rhee *et al.* (1999) – will be required in order to assess the possible contribution of the deletion genes to the overall process of human infection.

### Acknowledgements

TBLH is supported by a Wellcome Trust Medical Microbiology Fellowship (Grant number 053957/Z/98/Z). We thank Ms Monica Rebec and Mr Stuart Philip (St Mary's Hospital, London, UK) for their help in preparing the clinical isolates.

### References

Behr MA, Wilson MA, Gill WP, *et al.* 1999. Comparative genomics of BCG vaccines by whole-genome DNA microarray. *Science* **284**: 1520–1523.

Brosch R, Gordon SV, Eiglmeier K, *et al.* 2000. Genomics, biology, and evolution of the *Mycobacterium tuberculosis* complex. In *Molecular Genetics of Mycobacteria*, Hatfull GF, Jacobs WRJ (eds). ASM Press: Washington, DC; 19–36.

Brosch R, Philipp WJ, Stavropoulos E, Colston MJ, Cole ST, Gordon SV. 1999. Genomic analysis reveals variation between *Mycobacterium tuberculosis* H37Rv and the attenuated *M. tuberculosis* H37Ra strain. *Infect Immun* **67**: 5768–5774.

Chaves F, Yang Z, el Hajj H, *et al.* 1996. Usefulness of the secondary probe pTBN12 in DNA fingerprinting of *Mycobacterium tuberculosis*. *J Clin Microbiol* **34**: 1118–1123.

Cole ST, Brosch R, Parkhill J, *et al.* 1998. Deciphering the biology of *Mycobacterium tuberculosis* from the complete genome sequence. *Nature* **393**: 537–544.

Dye C, Scheele S, Dolin P, Pathania V, Raviglione MC. 1999. Consensus statement. Global burden of tuberculosis: estimated incidence, prevalence, and mortality by country. WHO Global Surveillance and Monitoring Project. *J Am Med Assoc* **282**: 677–686.

Fang Z, Doig C, Kenna DT, *et al.* 1999. IS*6110*-mediated deletions of wild-type chromosomes of *Mycobacterium tuberculosis*. *J Bacteriol* **181**: 1014–1020.

Fang Z, Forbes KJ. 1997. A *Mycobacterium tuberculosis* IS*6110* preferential locus (*ipl*) for insertion into the genome. *J Clin Microbiol* **35**: 479–481.

Fomukong N, Beggs M, el Hajj H, Templeton G, Eisenach K, Cave MD. 1997. Differences in the prevalence of IS*6110* insertion sites in *Mycobacterium tuberculosis* strains: low and high copy number of IS*6110*. *Tuberc Lung Dis* **78**: 109–116.

Gordon SV, Brosch R, Billault A, Garnier T, Eiglmeier K, Cole ST. 1999. Identification of variable regions in the genomes of tubercle bacilli using bacterial artificial chromosome arrays. *Mol Microbiol* **32**: 643–655.

Goyal M, Saunders NA, van Embden JD, Young DB, Shaw RJ. 1997. Differentiation of *Mycobacterium tuberculosis* isolates by spoligotyping and IS*6110* restriction fragment length polymorphism. *J Clin Microbiol* **35**: 647–651.

Kamerbeek J, Schouls L, Kolk A, *et al.* 1997. Simultaneous detection and strain differentiation of *Mycobacterium tuberculosis* for diagnosis and epidemiology. *J Clin Microbiol* **35**: 907–914.

Kurepina NE, Sreevatsan S, Plikaytis BB, *et al.* 1998. Characterization of the phylogenetic distribution and chromosomal insertion sites of five IS*6110* elements in *Mycobacterium tuberculosis*: non-random integration in the dnaA–dnaN region. *Tuberc Lung Dis* **79**: 31–42.

Mitchison DA, Selkon JB, Lloyd J. 1963. Virulence in the guinea pig, susceptibility to hydrogen peroxide, and the catalase activity of isoniazid-sensitive tubercle bacilli from South Indian and British patients. *J Pathol Bacteriol* **86**: 377–386.

Ordway DJ, Sonnenberg MG, Donahue SA, Belisle JT, Orme IM. 1995. Drug-resistant strains of *Mycobacterium tuberculosis* exhibit a range of virulence for mice. *Infect Immun* **63**: 741–743.

Rhee JT, Piatek AS, Small PM, *et al.* 1999. Molecular epidemiologic evaluation of transmissibility and virulence of *Mycobacterium tuberculosis*. *J Clin Microbiol* **37**: 1764–1770.

Sampson SL, Warren RM, Richardson M, van der Spuy GD, van Helden PD. 1999. Disruption of coding regions by IS*6110* insertion in *Mycobacterium tuberculosis*. *Tuberc Lung Dis* **79**: 349–359.

Schafer W. 1993. The role of cutinase in fungal pathogenicity. *Trends Microbiol* **1**: 69–71.

Small PM, Hopewell PC, Singh SP, *et al.* 1994. The epidemiology of tuberculosis in San Francisco. A population-based study using conventional and molecular methods. *N Engl J Med* **330**: 1703–1709.

Sreevatsan S, Pan X, Stockbauer KE, *et al.* 1997. Restricted structural gene polymorphism in the *Mycobacterium tuberculosis* complex indicates evolutionarily recent global dissemination. *Proc Natl Acad Sci U S A* **94**: 9869–9874.

Titball RW. 1993. Bacterial phospholipases C. *Microbiol Rev* **57**: 347–366.

Valway SE, Sanchez MP, Shinnick TF, *et al.* 1998. An outbreak involving extensive transmission of a virulent strain of *Mycobacterium tuberculosis*. *N Engl J Med* **338**: 633–639.

van Embden JD, Cave MD, Crawford JT, *et al.* 1993. Strain identification of *Mycobacterium tuberculosis* by DNA fingerprinting: recommendations for a standardized methodology. *J Clin Microbiol* **31**: 406–409.

Vera-Cabrera L, Howard ST, Laszlo A, Johnson WM. 1997. Analysis of genetic polymorphism in the phospholipase region of *Mycobacterium tuberculosis*. *J Clin Microbiol* **35**: 1190–1195.

Zhang Y, Mazurek GH, Cave MD, *et al.* 1992. DNA polymorphisms in strains of *Mycobacterium tuberculosis* analyzed by pulsed-field gel electrophoresis: a tool for epidemiology. *J Clin Microbiol* **30**: 1551–1556.

Copyright © 2000 John Wiley & Sons, Ltd. 
